# Enhancing spatial resolution in Fourier transform infrared spectral image via machine learning algorithms

**DOI:** 10.1038/s41598-023-50060-0

**Published:** 2023-12-20

**Authors:** Mina Lim, Kyu Ho Park, Jae Sung Hwang, Mikyung Choi, Hui Youn Shin, Hong-Kyu Kim

**Affiliations:** 1 Advanced Analysis and Data Center, Korea Institute of Science and Technology, Seoul, 02792 Republic of Korea; 2 School of Industrial and Management Engineering, Korea University, Seoul, 02841 Republic of Korea; 3 Materials and Devices Advanced Research Institute, LG Electronics, Seoul, 07796 Republic of Korea

**Keywords:** Characterization and analytical techniques, Imaging techniques

## Abstract

Owing to the intrinsic signal noise in the characterization of chemical structures through Fourier transform infrared (FT-IR) spectroscopy, the determination of the signal-to-noise ratio (SNR) depends on the level of the concentration of the chemical structures. In situations characterized by limited concentrations of chemical structures, the traditional approach involves mitigating the resulting low SNR by superimposing repetitive measurements. In this study, we achieved comparable high-quality results to data scanned 64 times and superimposed by employing machine learning algorithms such as the principal component analysis and non-negative matrix factorization, which perform the dimensionality reduction, on FT-IR spectral image data that was only scanned once. Furthermore, the spatial resolution of the mapping images correlated to each chemical structure was enhanced by applying both the machine learning algorithms and the Gaussian fitting simultaneously. Significantly, our investigation demonstrated that the spatial resolution of the mapping images acquired through relative intensity is further improved by employing dimensionality reduction techniques. Collectively, our findings imply that by optimizing research data through noise reduction enhancing spatial resolution using the machine learning algorithms, research processes can be more efficient, for instance by reducing redundant physical measurements.

## Introduction

Fourier-Transform Infrared Spectroscopy (FT-IR) is a widely used analytical method in the field of materials science. FT-IR analysis can reveal the molecular composition of the sample through infrared-induced vibrations of molecules. It is a popular method also since FT-IR measurements can be conducted across a wide sample area to discern chemical structures. However, variations in the experimental environment or limitations of the measurement equipment can introduce unnecessary noise to the spectral data, thereby diminishing the precision and reliability of the analysis results. To address these challenges, a frequent approach involves enhancing the image's spatial resolution. This is achieved by obtaining several spectral images of the same region and subsequently combining and averaging the outcomes^1–4^, by optimizing the IR micro-spectroscopy based on optical theory and mathematical models^5–11^, or by applying various signal processing algorithms such as Fourier transform (FT)^12^, wavelet transform (WT)^13^, Hilbert-Huang transform (HHT)^14^, minimum noise transform (MNT)^15–17^, and variational mode decomposition (VMD)^18^. While these techniques yield a high signal-to-noise ratio (SNR), it also requires significant instrument and human resources. To optimize measurement efficiency, it is crucial to employ a methodology that reduces the number of redundant measurements and simplifies the equipment calibration process while simultaneously enhancing the precision of the outcomes.

To address this, recent advances in artificial intelligence have led to attempts to improve the spatial resolution of spectral images acquired by scanning electron microscope (SEM) equipped with energy dispersive X-ray spectroscopy (EDS) and scanning transmission electron microscope (STEM) equipped with EDS or electron energy loss spectroscopy (EELS) using machine learning algorithms^19–23^. Potapov et al.^19,20^ achieved enhanced spatial resolution in STEM-EDS mapping images by employing principal component analysis (PCA) for noise reduction. Similarly, Kim et al.^21^ employed a combination of PCA and independent component analysis (ICA) to significantly improve the spatial resolution of STEM-EDS, allowing for the observation of previously undetected depletion regions in light elements after data processing. Teng and Gauvin investigated the application of the non-negative matrix factorization (NMF) algorithm to SEM–EDS spectral image data for the purpose of phase classification in rare earth minerals^22^, and Muto and Shiga exploited the NMF algorithm to enhance spatial resolution by mitigating noise in STEM-EELS spectral image data^23^. A common thread in these previous studies is the achievement of notable enhancements in the SNR through the application of dimensionality reduction algorithms such as PCA, ICA and NMF. These enhancements have consequently led to an augmented spatial resolution in spectral images. In the context of FT-IR spectroscopy, recent studies have focused on the utilization of deep learning or machine learning algorithms to classify individual molecular structures within FT-IR spectral images^24–26^. However, as previously noted, there is a necessity to develop a methodology that could enhance the spatial resolution of FT-IR spectral images without the involvement of humans.

In this paper, we introduce an approach aimed at enhancing spatial resolution by mitigating noise present in spectral images acquired via FT-IR measurements, employing machine learning algorithms. Our approach focuses on the efficiency of simple data processing by comparing data with increased spatial resolution to data with dozens of repeated measurements. Furthermore, we elucidate a technique involving Gaussian model fitting, which proves to be an efficient means of visualizing mapping images that highlight specific molecular structures within the spectral data.

## Methods

### Sample preparation

The sample composition was prepared by blending 20 wt% of 7–10 µm sized Polytetrafluoroethylene (PTFE) powder with 80 wt% of Polyamide-imide (PAI). The PTFE powder was introduced into a PAI solvent and subjected to ball milling at room temperature for a duration of one hour. Subsequently, the resultant mixture was applied onto an aluminum substrate using the spray coating technique. The coated substrate was then subjected to a drying process at 220 ℃, resulting in the formation of an 80 µm thick PTFE coating film.

### Noise reduction

To reduce noise and achieve a high signal-to-noise ratio (SNR) in FT-IR spectral image data, we employed dimensionality reduction algorithms, specifically singular value decomposition (SVD) and non-negative matrix factorization (NMF). To initiate denoising through dimensionality reduction, the dataset needed to be first transformed into a matrix, denoted as M, with spatial dimensions of 32 × 32 and an energy dimension of 1506. We began by performing the SVD algorithm to analyze eigenvalues and principal components. SVD decomposes the data matrix $M$ as follows:

$$M = U\sum VT$$

here $M$ has dimensions 1024 × 1506, where $U$ and $V$ are the left and right singular vector matrices, respectively, and $\sum$ is a diagonal matrix containing the singular values of $M$. Notably, the diagonal entries of $\sum$ represent eigenvalues, which serve as the basis for establishing a threshold for subsequent NMF processing. Subsequently, we employed the NMF algorithm, a dimensionality reduction machine learning algorithm well-suited for non-negative matrices. Given that FT-IR spectral image data consists of positive values, NMF was chosen as it demonstrated the best performance. NMF effectively decomposes $M$ as follows:

$$M \approx WH$$

Leveraging the threshold established through the SVD analysis, we proceeded to extract features from $WH$. This process culminated in the reconstruction of de-noised FT-IR mapping images.

### Gaussian model fitting

The noise reduction effect is enhanced by utilizing the Gaussian model fitting method at each peak position to calculate the relative intensity. Gaussian model fitting involves optimizing parameters such as amplitude, sigma, and offset using Gaussian functions. To obtain the level of the relative intensity at the desired peaks, a predefined range is set on both sides of the peak, and the amplitude, sigma, and offset values are refined using the least-squares function. This process is applied to all spectral points in spatial dimensions, resulting in a more prominent reconstruction of denoised FT-IR mapping images.

## Results and discussion

**Figure 1:**
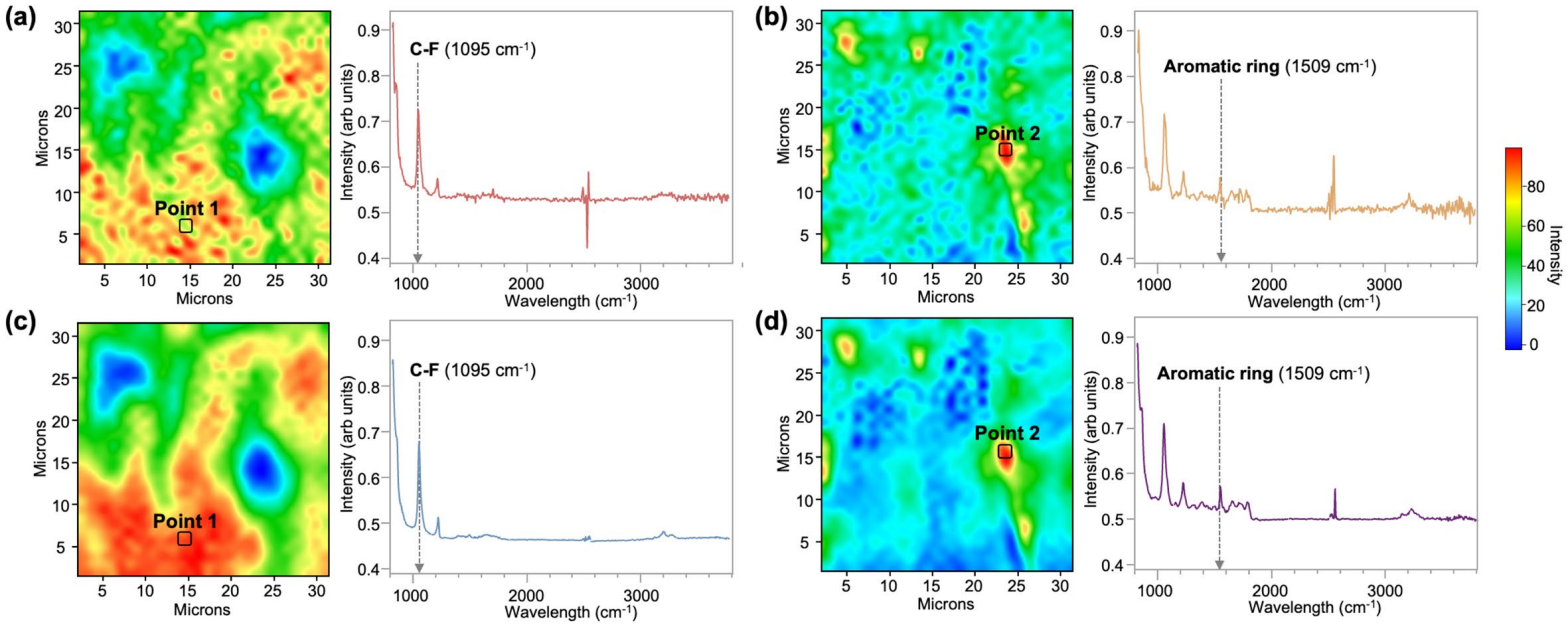
Experimental mapping images and spectra of C–F bonding and aromatic ring structures. Extracted from a single scan: (**a**) mapping image and spectrum for C–F bond structure, (**b**) those for aromatic ring structure. Extracted from 64 scans and averaged: (**c**) mapping image and spectrum for C–F bond structure, (**d**) those for aromatic ring structure. Notably, the Point 1 and 2 that are denoted the mapping images correspond to the same spatial locations in both the single and 64-time scan datasets.

Figure 1 provides a demonstration of how repeated FT-IR measurements can enhance spatial resolution. Figure 1a presents the spectrum of a single-scan spectral image, while Figs. 1b,c display mapping images underlining wavelengths corresponding to C–F bond molecules and aromatic rings. Notably, the fabricated material exhibits a heterogeneous composition, with the coating layer comprising a mixture of PTFE and PAI. In the results of a single scan, distinct peaks representative of each molecular structure within the material are discernible mostly. Nonetheless, the presence of noise generated during the scanning process hinders the distinct differentiation between the PTFE and PAI material regions within the mapping images illustrating C–F bonding molecules (Fig. 1a) and aromatic ring structures (Fig. 1b). These regions are typically characterized by the areas exhibiting the highest intensity in each respective mapping image. Typically, the low spatial resolution inherent in these spectral images is addressed by averaging the results of repeated measurements. Indeed, the spectrum obtained by averaging 64 repetitions of measurements on the same sample (Fig. 1c,d) illustrates the efficiency of this approach in yielding high SNR data. Regarding the mapping image illustrating the distribution of C–F bond molecules, it is noteworthy that the high-intensity regions observed in the single scan result (Fig. 1a) become more conspicuously defined in the dataset derived from 64 repeated scans (Fig. 1c). This improvement in spatial resolution through repeated measurements is consistently perceived in the mapping image of the aromatic ring structure (Fig. 1b,d). Nonetheless, the pursuit of high spatial resolution measurements inherently involves resource-intensive processes such as dozens of repeated measurements, demanding a technique to minimize such resource consumption.

**Figure 2:**
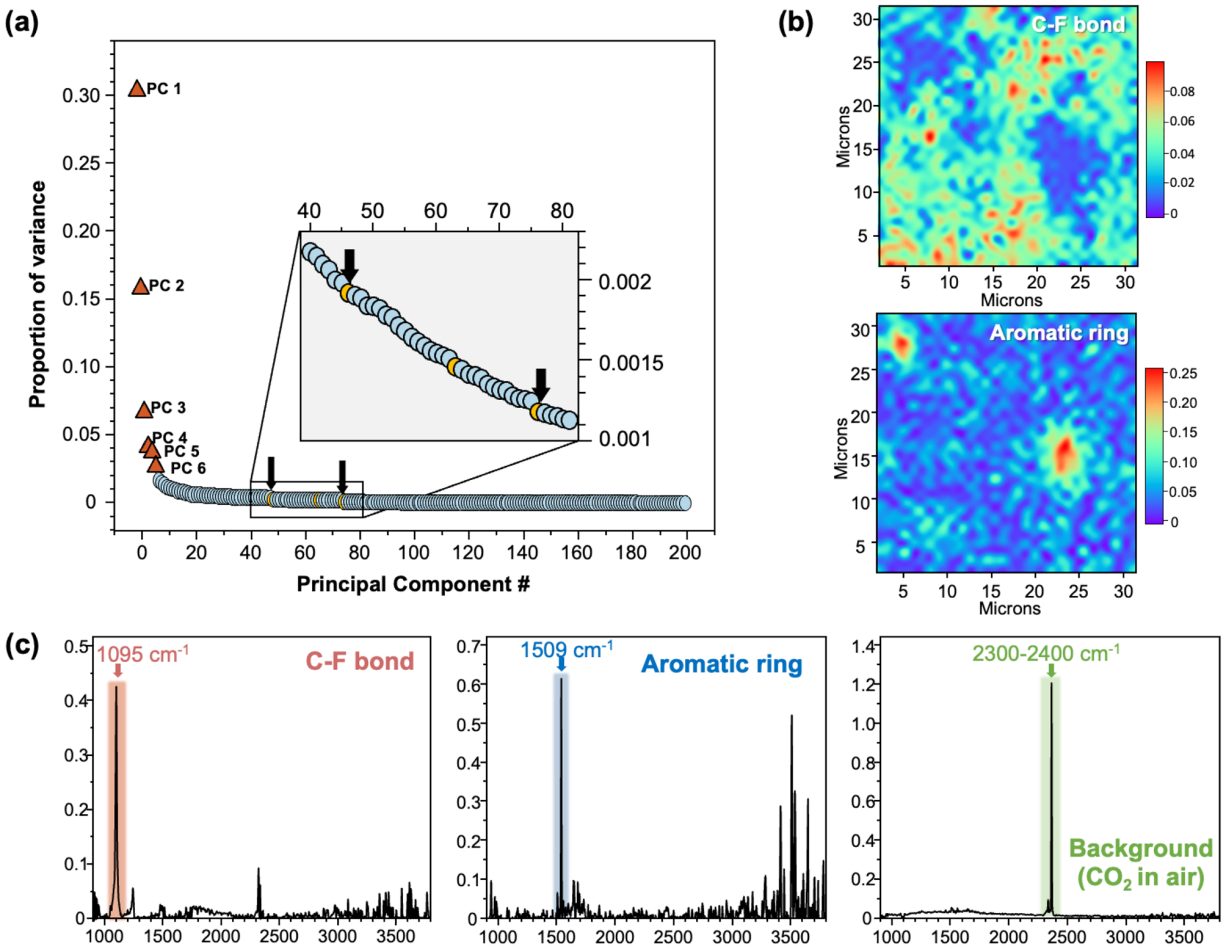
Component decomposition by applying NMF algorithm to spectral image data. (**a**) the scree plot, (**b**) maps (loadings) and (**c**) signals (factors) of primary components.

Dimensionality reduction techniques, specifically singular value decomposition (SVD) and NMF, were employed to reduce noise within a single-scanned spectral image, thereby enhancing spatial resolution efficiently. An initial step involved decomposing the spectral image elements using the SVD algorithm. The determination of how many of principal components should be recombined to achieve dimensionality reduction was crucial. To address this, SVD was performed on a once-scanned spectral image, and the scree plot illustrating the eigenvalues of variance for each component is depicted in Fig. 2a. Commonly, when conducting dimensionality reduction for noise mitigation, the variation on the scree plot, where the slope changes significantly, serves as the criterion for dimensionality reduction^27–30^. However, the target substances for dimensionality reduction are the C–F bond and aromatic ring structures observed in the mixed sample of PTFE and PAI, and the C–F bond structure is measured with high intensity and is expected to be decomposed into a relatively small number (corresponding to high eigenvalue) of principal components, while the aromatic ring structure is measured with very low intensity at the noise level and is expected to be decomposed into a relatively large number of principal components (low eigenvalue). Consequently, in the interest of selecting components that capture subtle eigenvalue variations, the decision was made to include components where the sharp drop in eigenvalue ceased to occur, as demonstrated in the inset of Fig. 2a. The mapping images illustrates the loading of the selected components, revealing the decomposition into C–F bond structure and aromatic ring, as shown in Fig. 2b. These phenomena are also observed in the score matrix of each component (Fig. 2c), which shows that each signal is decomposed into C–F bond, aromatic ring structure and background. Notably, the symmetric and asymmetric stretching peaks of the C–F bond structure were grouped into the same component because both peaks were always observed simultaneously, indicating that the decomposition algorithm was well adapted to the phase decomposition of the material.

**Figure 3:**
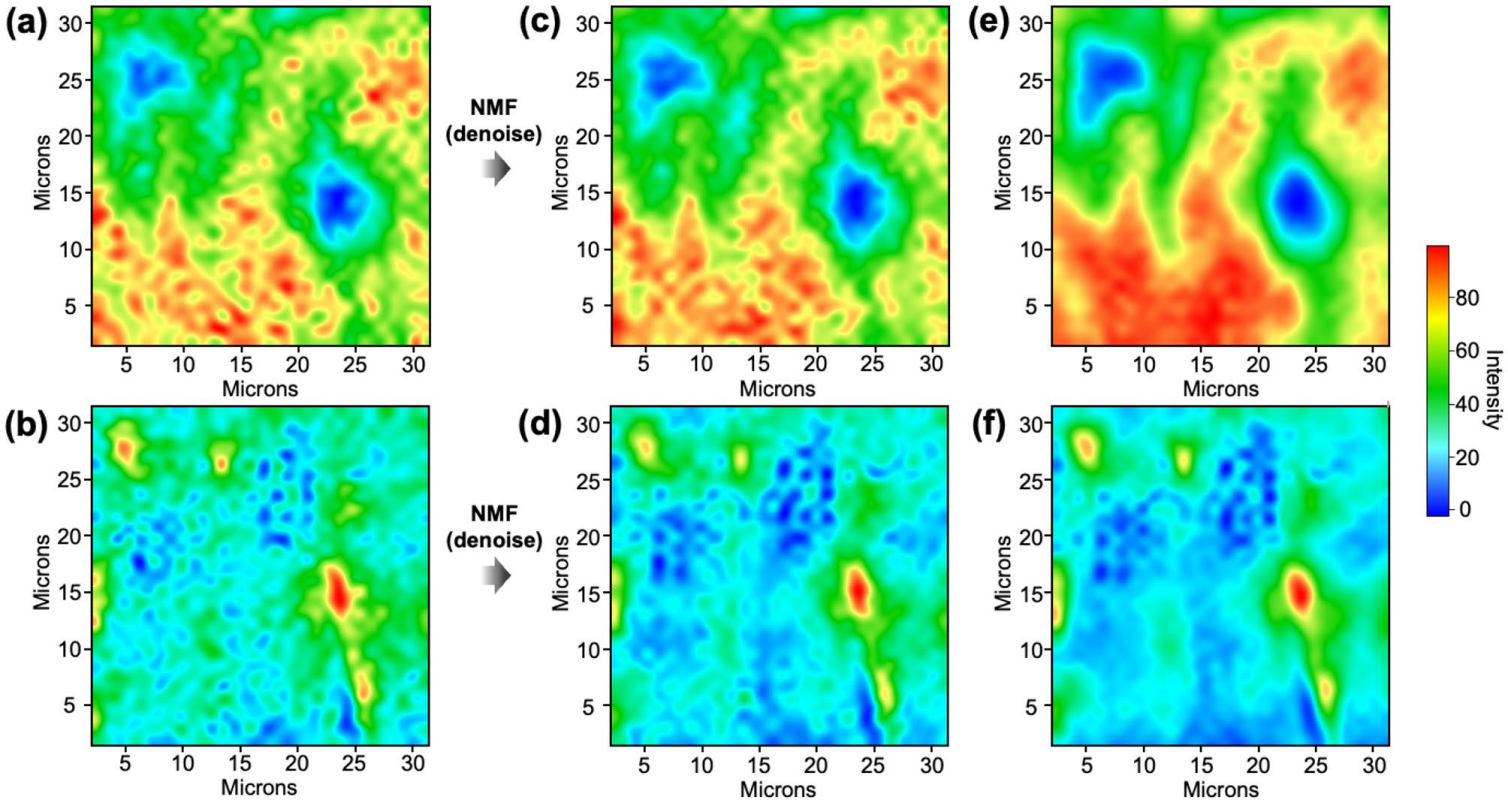
Improvement in spatial resolution by data processing of a single-scan spectral image and comparison with data from 64 repeated scans. Mapping images of (**a**) C–F bonds and (**b**) aromatic ring molecules extracted from single-scan data, (**c**) C–F bonds and (**d**) aromatic ring molecules extracted after application of NMF, and (**e**) C–F bonds and (**f**) aromatic ring molecules extracted from data scanned 64 times.

The mapping images of the C–F bond and aromatic ring structures, derived from the raw data scanned only once, exhibit relatively low spatial resolution primarily due to high noise levels (Fig. 3a,b). Remarkably, the intensity at the wavelength of 1509 cm^−1^, which represents the aromatic ring in the measured spectrum, is exceedingly low, rendering it challenging to differentiate from the noise. Thus, the mapping image describing the distribution of the aromatic ring structure appears indistinct (Fig. 3b). To address this limitation, the raw data obtained from the instrument was subjected to the NMF algorithm to mitigate noise and enhance spatial resolution. The resulting mapping images of the C–F bond and aromatic ring molecules are presented in Fig. 3c,d. The mapping image depicting the C–F bond structure with high intensity, interestingly, remained relatively unchanged before and after the application of the NMF algorithm. In contrast, the mapping image representing the aromatic ring structure, characterized by relatively low intensity and difficulty in distinguishing it from noise, exhibited a dramatic enhancement in spatial resolution following NMF (Fig. 3d). This phenomenon stems from the character that the degree of enhancement in spatial resolution achieved through dimensionality reduction is contingent upon the intensity of individual peaks; the peak associated with the C–F bond structure possesses significantly higher intensity compared to the prevailing noise level, resulting in a limited effect on the SNR improvement through noise reduction. Conversely, the peak indicative of the aromatic ring in the original data is challenging to discern from noise, leading to a pronounced effect of SNR improvement following noise reduction. This phenomenon aligns with findings reported in prior studies^3–7^ focusing on resolution enhancement through dimensionality reduction techniques such as PCA, ICA, and NMF.

To validate the improvement of the spatial resolution, the mapping images achieved through the simple machine learning processing were compared with those achieved through the superimposition of 64 repeated scans. The mapping images of the C–F bond and aromatic ring structures (Fig. 3e,f), which were generated by averaging the results of repeated measurements of the same sample area, distinctly reveal the spatial distribution of each structure compared to the results obtained from a single scan. Comparing the mapping images derived from repeated measurements with the results of the spatial resolution enhancement obtained by post-processing data from a single scan, it is evident that the spatial resolution enhancement obtained by the machine learning algorithm is relatively consistent with the repeated measurements. Particularly, the distribution of regions corresponding to the C–F bond structure with high and low intensity, shown in red and blue respectively in Fig. 3, becomes similar to the data scanned 64 times. This trend is similarly observed in the mapping image representing the aromatic ring structure.

**Figure 4:**
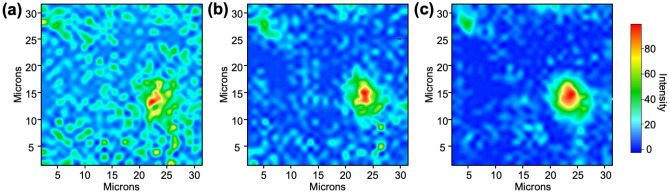
Enhanced spatial resolution via Gaussian model fitting. Aromatic ring molecule mapping images (**a**) before and (**b**) after applying Gaussian model fitting to the resulting spatial resolution enhancement by the NMF algorithm, and (**c**) after applying Gaussian model fitting to data scanned experimentally 64 times.

The noise reduction effect of dimensionality reduction manifests more prominently in mapping results with the relative height of the intensity calculated using Gaussian model fitting method at each peak position compared to those with the absolute height of the intensity. Figure 4a illustrates the mapping result of the aromatic ring structure, reconstructed from the data presented in Fig. 3a, applying the relative height of the intensity via Gaussian model fitting. In this result, the distribution of the aromatic ring structure appears widely dispersed. However, when examining the mapping result in Fig. 4b, derived from a single scan of these reconstructed data, followed by noise reduction through the simple processing, it is evident that islands of aromatic ring structures emerge in the upper-left and middle-right regions of the image. Given the aggregating nature of the aromatic ring molecules^14^, it seems more plausible that these islands have formed, as depicted in Fig. 4b, rather than a well-dispersed result resembling Fig. 4a. This conjecture finds support in the superimposed results of 64 scans presented in Fig. 4c. The mapping with the relative intensities, calculated via Gaussian model fitting from experimentally acquired and superimposed results, obviously reveals that the aromatic ring structure is distributed in the form of small islands in the upper-left and large islands in the middle-right, which is consistent with the phenomena described above. In other words, similar to the results in Fig. 3, the mapping results with Gaussian model fitting also indicate that dimensionality reduction by the machine learning algorithm can effectively remove noise. Notably, in contrast to the result shown in Fig. 3, where each point in the spectral image shared a consistent spectral baseline, it becomes evident that relying on the relative height of the peak intensity, based on Gaussian model fitting, proves more effective in elucidating structural details than relying solely on the absolute intensity for mapping a specific structure.

We quantitatively compare the noise reduction capabilities of our algorithm to several previously reported spatial resolution enhancement algorithms in spectroscopy^12–14^, as shown in Table 1. Traditional signal processing algorithms, such as FT^12^, WT^13^, and HHF^14^ algorithms, were used to decompose a spectrum into intrinsic mode functions and perform noise reduction and spatial resolution enhancement. For quantitative comparison, the degree of the spatial resolution enhancement was calculated using the structural similarity index measurement (SSIM)^31^. When comparing the mappings of C–F bonding and aromatic ring images obtained from the 64-scanned results with those obtained from the 1-scanned results that underwent noise reduction using FT, WT, HHT, and NMF algorithms, the NMF algorithm exhibited better noise reduction performance than the alternative FT, WT, and HHT algorithms in all cases. From this perspective, the presented NMF method is the most suitable algorithm for noise reduction and spatial resolution enhancement in FT-IR spectral images. Additionally, the proposed algorithm has the advantage of convenience over other methods. The results can be obtained promptly by importing raw data from the measured FT-IR spectra images without requiring any human intervention, such as defining threshold values.

**Table 1:** Quantitative comparison of spatial resolution enhancement performance of different algorithms with SSIM.

	1st scanned	FT^12^	WT^13^	HHT^14^	Proposed method
C–F bonding (1095 cm^2^)	0.998017	0.998158 (+ 0.014%)	0.998905 (+ 0.089%)	0.921744 (− 7.642%)	0.999106 (+ 0.109%)
Aromatic ring (1509 cm^2^)	0.996624	0.9967703 (+ 0.015%)	0.999395 (+ 0.278%)	0.912381 (− 8.453%)	0.999887 (+ 0.327%)

All data were compared to mapping images obtained from the experimental result scanned and superimposed with 64 times.

## Conclusions

By combining unsupervised machine learning algorithms such as SVD and NMF, the effective noise reduction can be achieved through dimensionality reduction of FT-IR spectral image data, which can dramatically reduce the frequency of physical measurements, which are typically repeated dozens of times to improve SNR. In the composite sample composed of PTFE and PAI, vital indicators such as the distribution of C–F bond and aromatic ring structures exhibited notable similarities after undergoing data processing via machine learning algorithms in comparison to the mapping image derived directly from the raw data. Notably, due to the noise reduction process, the C–F bond structure, characterized by a high SNR even in the unprocessed raw data owing to its relatively high intensity, experienced minimal dimensionality reduction effects. Conversely, the aromatic ring structure, measured with low intensity that made it challenging to distinguish from noise, exhibited a profound dimensionality reduction effect through data processing. In conclusion, the incorporation of data processing utilizing computational power is suggested as a means to minimize the repetitive measurement procedures in FT-IR, typically employed to achieve high SNR. Studies on spatial resolution enhancement using a deep learning approach for spectral images have been reported recently^32–36^, and it is valuable to explore and compare the performance of signal processing, multivariate analysis, and deep learning algorithms for improving the spatial resolution of FT-IR spectral images, which we will explore in future work.

## Data Availability

The datasets used and/or analysed during the current study available from the corresponding author on reasonable request.

## References

[1] Snively CM, Koenig JL (1998). Characterizing the performance of a fast FT-IR imaging spectrometer. Appl. Spectrosc..

[2] Rammelsberg R, Boulas S, Chorongiewski H, Gerwert K (1999). Set-up for time-resolved step-scan FTIR spectroscopy of noncyclic reactions. Vib. Spectrosc..

[3] Bhargava R, Levin IW (2001). Fourier transform infrared imaging: Theory and practice. Anal. Chem..

[4] Barra I, Khiari L, Haefele SM, Sakrabani R, Kebede F (2021). Optimizing setup of scan number in FTIR spectroscopy using the moment distance index and PLS regression: Application to soil spectroscopy. Sci. Rep..

[5] Davis BJ, Carney PS, Bhargava R (2010). Theory of midinfrared absorption microspectroscopy: I. Homogeneous samples. Anal. Chem..

[6] Davis BJ, Carney PS, Bhargava R (2010). Theory of mid-infrared absorption microspectroscopy: II. Heterogeneous samples. Anal. Chem..

[7] Reddy R, Mayerich D, Walsh M, Schulmerich M, Carney PS, Bhargava R (2012). Optimizing the design of FT-IR spectroscopic imaging instruments to obtain increased spatial resolution of chemical species. 2012 9th IEEE Int. Symp. Biomed. Imaging (ISBI).

[8] Reddy RK, Walsh MJ, Schulmerich MV, Carney PS, Bhargava R (2012). High-definition infrared spectroscopic imaging. Appl. Spectrosc..

[9] Chan KLA, Kazarian SG (2013). Correcting the effect of refraction and dispersion of light in FT-IR spectroscopic imaging in transmission through thick infrared windows. Anal. Chem..

[10] Rasskazov IL, Singh R, Carney PS, Bhargava R (2019). Extended multiplicative signal correction for infrared microspectroscopy of heterogeneous samples with cylindrical domains. Appl. Spectrosc..

[11] Phal Y, Pfister L, Carney PS, Bhargava R (2022). Resolution limit in infrared chemical imaging. J. Phys. Chem. C..

[12] Wahab MF, Gritti F, O’Haver TC (2021). Discrete Fourier transform techniques for noise reduction and digital enhancement of analytical signals. TrAC Trends Anal. Chem..

[13] Shao X-G, Leung AK-M, Chau F-T (2003). Wavelet: A new trend in chemistry. Acc. Chem. Res..

[14] Bian X, Ling M, Chu Y, Liu P, Tan X (2022). Spectral denoising based on Hilbert-Huang transform combined with F-test. Front. Chem..

[15] Bhargava R, Ribar T, Koenig JL (1999). Towards faster FT-IR imaging by reducing noise. Appl. Spectrosc..

[16] Bhargava R, Wang S-Q, Koenig JL (1999). Route to higher fidelity FT-IR imaging. Appl. Spectrosc..

[17] Reddy RK, Bhargava R (2010). Accurate histopathology from low signal-to-noise ratio spectroscopic imaging data. Analyst.

[18] Bian X, Shi Z, Shao Y, Chu Y, Tan X (2023). Variational mode decomposition for raman spectral denoising. Molecules.

[19] Potapov P, Longo P, Okunishi E (2017). Enhancement of noisy EDX HRSTEM spectrum-images by combination of filtering and PCA. Micron.

[20] Potapov P, Lubk A (2019). Optimal principal component analysis of STEM XEDS spectrum images. Adv. Struct. Chem. Imaging.

[21] Kim H-K, Ha H-Y, Bae J-H, Cho MK, Kim J, Han J, Suh J-Y, Kim G-H, Lee T-H, Jang JH, Chun D (2020). Nanoscale light element identification using machine learning aided STEM-EDS. Sci. Rep..

[22] Teng C, Gauvin R (2020). Multivariate statistical analysis on a SEM/EDS phase map of rare earth minerals. Scanning.

[23] Muto S, Shiga M (2019). Application of machine learning techniques to electron microscopic/spectroscopic image data analysis. Microscopy.

[24] Lasch P, Stämmler M, Zhang M, Baranska M, Bosch A, Majzner K (2018). FT-IR hyperspectral imaging and artificial neural network analysis for identification of pathogenic bacteria. Anal. Chem..

[25] Raczkowska MK, Koziol P, Urbaniak-Wasik S, Paluszkiewicz C, Kwiatek WM, Wrobel TP (2019). Influence of denoising on classification results in the context of hyperspectral data: High definition FT-IR imaging. Anal. Chim. Acta..

[26] Liu Y, Yao W, Qin F, Zhou L, Zheng Y (2023). Spectral classification of large-scale blended (Micro) plastics using FT-IR raw spectra and image-based machine learning. Environ. Sci. Technol..

[27] Schanze T (2018). Compression and noise reduction of biomedical signals by singular value decomposition. IFAC-PapersOnLine.

[28] Ozawa K (2023). Noise reduction of low-count STEM-EDX data by low-rank regularized spectral smoothing. Microsc. Microanal..

[29] Lichtert S, Verbeeck J (2013). Statistical consequences of applying a PCA noise filter on EELS spectrum images. Ultramicroscopy.

[30] Gómez-Hortigüela L, Hamad S, López-Arbeloa F, Pinar AB, Pérez-Pariente J, Corà F (2009). Molecular insights into the self-aggregation of aromatic molecules in the synthesis of nanoporous aluminophosphates: A multilevel approach. J. Am. Chem. Soc..

[31] Wang Z, Bovik AC, Sheikh HR, Simoncelli EP (2004). Image quality assessment: From error visibility to structural similarity. IEEE Trans. Image Process..

[32] Horgan CC, Jensen M, Nagelkerke A, St-Pierre J-P, Vercauteren T, Stevens MM, Bergholt MS (2021). High-throughput molecular imaging via deep-learning-enabled raman spectroscopy. Anal. Chem..

[33] Falahkheirkhah K, Yeh K, Mittal S, Pfister L, Bhargava R (2021). Deep learning-based protocols to enhance infrared imaging systems. Chemom. Intell. Lab. Syst..

[34] Chatzidakis M, Botton GA (2019). Towards calibration-invariant spectroscopy using deep learning. Sci Rep-UK..

[35] Juntunen C, Woller IM, Abramczyk AR, Sung Y (2022). Deep-learning-assisted Fourier transform imaging spectroscopy for hyperspectral fluorescence imaging. Sci. Rep..

[36] Ziatdinov M, Ghosh A, Wong CY, Kalinin SV (2022). AtomAI framework for deep learning analysis of image and spectroscopy data in electron and scanning probe microscopy. Nat. Mach. Intell..

